# A geometry‐based scaling model for stereotactic treatment volume definition in stereotactic centralized ablative radiation therapy

**DOI:** 10.1002/acm2.70713

**Published:** 2026-07-26

**Authors:** Ningjing Siah, Pengyuan Qi, Bin Hu, Mi Chen, Ting Cao, Xin Li, Jun Han

**Affiliations:** ^1^ Cancer Center, Union Hospital, Tongji Medical College Huazhong University of Science and Technology Wuhan China; ^2^ Institute of Radiation Oncology, Union Hospital, Tongji Medical College Huazhong University of Science and Technology Wuhan China; ^3^ Hubei Key Laboratory of Precision Radiation Oncology Wuhan China; ^4^ Department of Radiation Oncology The Second People's Hospital of Yichang The Second People's Hospital of China Three Gorges University Yichang Hubei China

**Keywords:** spatially fractionated radiotherapy, stereotactic centralized ablative radiation therapy (SCART), stereotactic treatment volume, target volume definition, VMAT

## Abstract

**Background:**

Stereotactic Centralized Ablative Radiation Therapy (SCART) is a spatially fractionated radiotherapy strategy designed for large or bulky tumors by delivering an intensified ablative dose to a centrally located subvolume while allowing controlled dose reduction toward the tumor periphery. However, clinical implementation of SCART critically depends on reproducible definition of the stereotactic treatment volume (STV), which currently lacks a standardized geometric framework.

**Purpose:**

To develop and preliminarily evaluate a geometry‐based scaling model for STV definition based on internal dose fall‐off characteristics under stereotactic volumetric modulated arc therapy (VMAT) delivery.

**Methods:**

A phantom‐based stereotactic VMAT planning study was performed using idealized cylindrical STVs with radii of 1–3 cm and axial lengths of 3–10 cm. VMAT plans were generated using prescription doses of 15–24 Gy per fraction over three fractions. Internal dose fall‐off was quantified using the equivalent radius (*r*_15_) of the 15 Gy isodose surface, defined as the radius of a volume‐equivalent cylindrical isodose distribution. The predicted STV radius (*d*) was subsequently modeled from outer reference target geometry using dose‐specific linear regression analysis.

**Results:**

The extracted 15 Gy isodose surface demonstrated reproducible geometry‐associated attenuation behavior across the investigated stereotactic VMAT configurations. The equivalent radius (*r*_15_) increased monotonically with STV size, ranging from approximately 2.0–2.1 cm to 4.1–4.3 cm at 15 Gy × 3 and from approximately 3.0–3.3 cm to 6.3–6.6 cm at 24 Gy × 3 as STV radius increased from 1 to 3 cm. Variation associated with axial target length generally remained within approximately 0.2–0.4 cm for a given prescription level. Increasing arc number produced only modest effects on dose attenuation behavior, with a median inter‐configuration difference in *r*_15_ of approximately 0.12 cm. Dose‐specific regression models demonstrated excellent agreement with phantom‐derived measurements, with R^2^ values ranging from 0.991 to 0.999.

**Conclusion:**

This study establishes a geometry‐based STV scaling framework for SCART based on reproducible internal dose fall‐off characteristics under stereotactic VMAT delivery. The proposed dose‐specific regression model provides a practical and physically interpretable approach for inward STV scaling from measurable gross tumor volume geometry and may support more reproducible implementation of SCART planning.

## 1 INTRODUCTION

Radiotherapy for large or bulky tumors remains challenging because dose escalation is often limited by surrounding organs at risk (OARs), particularly in centrally hypoxic tumor regions.^1^, ^2^, ^3^, ^4^, ^5^ Although approaches such as stereotactic hypofractionation, dose painting, and adaptive radiotherapy have been explored,^6^, ^7^, ^8^ reproducible delivery of ablative central dose while maintaining peripheral sparing remains difficult in clinical practice.

Stereotactic Centralized Ablative Radiation Therapy (SCART) is a spatially fractionated stereotactic strategy that delivers an ablative dose to a centrally located subvolume while allowing controlled peripheral dose reduction.^9^, ^10^, ^11^ Clinical implementation of SCART depends critically on reproducible definition of the stereotactic treatment volume (STV), which currently lacks a standardized geometric framework and remains largely dependent on subjective planner interpretation.^12^, ^13^

However, spatial dose fall‐off in stereotactic radiotherapy is influenced by multiple factors, including treatment platform characteristics, beam arrangement, modulation complexity, tissue heterogeneity, and target geometry.^14^, ^15^, ^16^ Under controlled stereotactic VMAT conditions, steep and spatially continuous internal dose attenuation patterns may nevertheless generate reproducible geometry‐associated relationships between target size and intermediate‐dose distribution within the investigated delivery configuration. Characterizing these relationships may therefore provide a practical basis for systematic inward STV scaling from an outer reference target.

Therefore, this study aimed to develop a clinically applicable and reproducible framework for STV definition in SCART. Using phantom‐based stereotactic VMAT simulations, we quantified the spatial behavior of internal dose fall‐off and derived dose‐specific models linking target geometry and prescription dose to inward STV scaling. This work provides practical model coefficients and a reproducible geometry‐guided workflow for prospective STV generation to support more standardized implementation of SCART in clinical practice.

## 2 MATERIALS AND METHODS

### 2.1 Study design and conceptual framework

A phantom‐based dosimetric study was designed to characterize internal dose fall‐off under stereotactic VMAT delivery. The analysis focused on the radial extent of the 15 Gy isodose surface relative to the prescribed STV boundary, quantified using an equivalent‐radius formulation.

**FIGURE 1:**
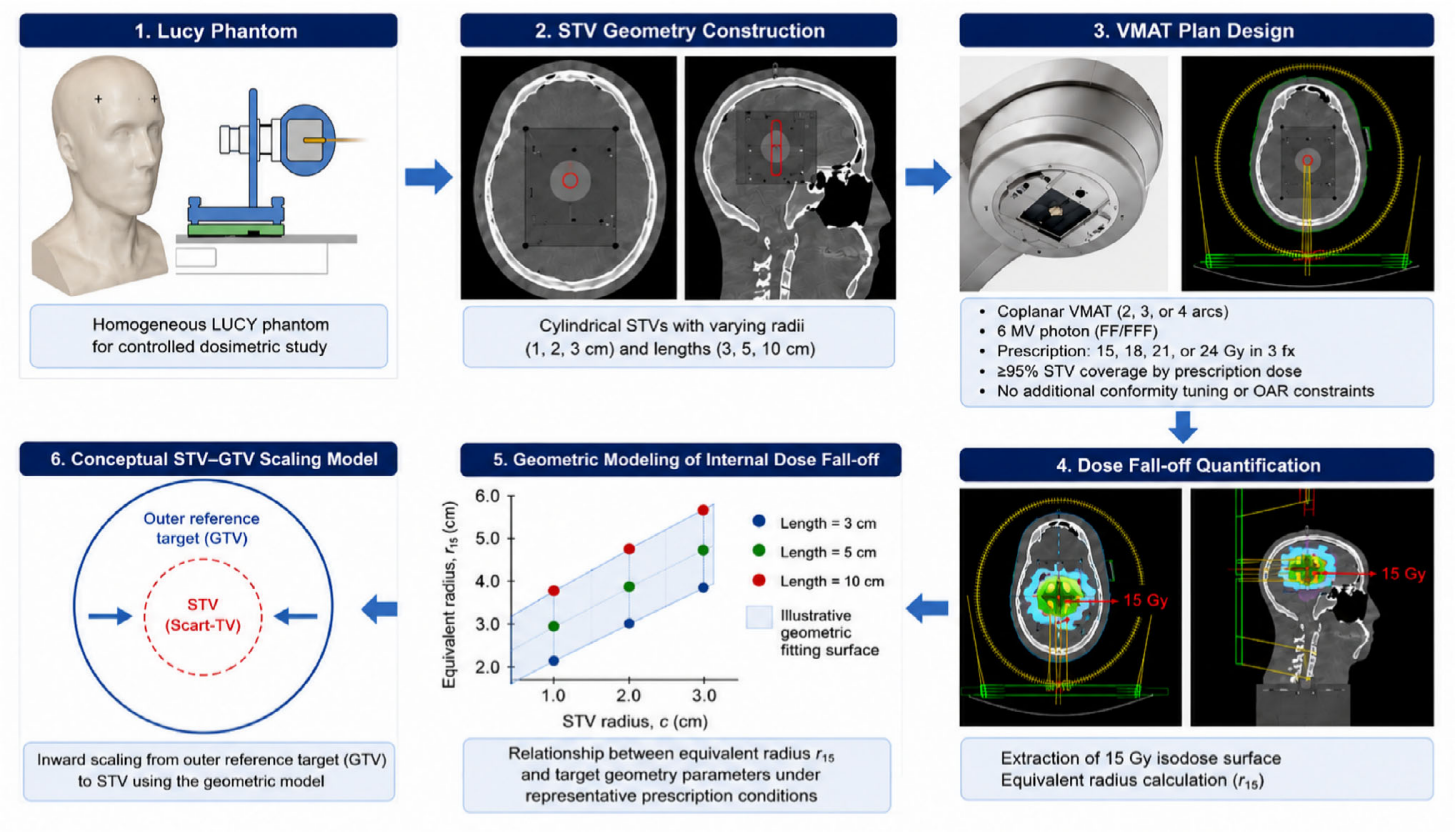
Overview of the study workflow and modeling framework. Schematic illustration of the phantom‐based workflow used for geometry‐based STV modeling for SCART, including STV geometry construction on the LUCY phantom, stereotactic VMAT plan generation, dose fall‐off quantification using the 15 Gy isodose surface, geometric modeling of internal dose attenuation, and derivation of a geometry‐based scaling model linking an outer reference target to the predicted STV radius and subsequent STV generation. The geometric fitting surface shown in Panel 5 is illustrative and intended only to demonstrate the conceptual scaling relationship. STV = stereotactic treatment volume; GTV = gross tumor volume; VMAT = volumetric modulated arc therapy; FF = flattening filter; FFF = flattening filter‐free; fx = fraction.

Idealized cylindrical STVs were used to minimize anatomic variability and isolate geometry‐associated dose attenuation behavior under controlled planning conditions. Because such simplified geometries do not fully represent irregular clinical tumors, the proposed model should be interpreted as a geometry‐based planning approximation rather than an exact predictor of patient‐specific dose distributions. The overall workflow is shown in Figure 1.

### 2.2 Phantom model and target geometry

The outer reference target is a purely geometric construct inferred from the intermediate isodose surface and does not represent an explicitly contoured clinical GTV. All simulations were conducted using the LUCY phantom to provide a homogeneous and reproducible dosimetric environment. This phantom‐based setup minimized the confounding effects of tissue heterogeneity and anatomical variability, thereby enabling isolation and evaluation of the intrinsic physical characteristics of VMAT dose distributions.

Within the phantom, a series of idealized cylindrical target volumes were constructed to serve as surrogates for the STV, representing the centrally prescribed high dose region in SCART. Cylindrical STVs were generated with radii of 1.0, 2.0, and 3.0 cm (corresponding diameters of 2, 4, and 6 cm) and axial lengths of 3, 5, or 10 cm, resulting in multiple combinations of target geometry. This geometric range was selected to represent clinically relevant centrally boosted stereotactic target dimensions commonly encountered in SCART‐style planning for bulky tumors while maintaining stable stereotactic VMAT dose gradients within the phantom framework. This systematic variation allowed independent assessment of radial and longitudinal dimensions on internal dose attenuation.

A cylindrical geometry was selected because its axial symmetry facilitates robust characterization of radial dose fall‐off and simplifies geometric interpretation. In this study, the cylindrical structures were used solely to represent the STV. The GTV was not explicitly contoured; instead, an outer reference target was inferred indirectly from the spatial characteristics of intermediate isodose surfaces.

### 2.3 Treatment planning and irradiation parameters

Coplanar VMAT plans were generated on Trilogy and Halcyon systems (Varian Medical Systems, Palo Alto, CA, USA) using 6 MV photon beams. For Trilogy, both flattening‐filter (FF) and flattening‐filter‐free (FFF) modes were evaluated, whereas Halcyon was operated in FFF mode. Plans were generated using two, three, or four coplanar arcs.

The STV was prescribed 15, 18, 21, or 24 Gy per fraction over three fractions. Prescription dose was treated as a stratification variable, and separate models were derived for each dose level. All plans were required to achieve ≥95% STV coverage by the prescription dose. Optimization objectives, planning priorities, and dose calculation settings were applied consistently across configurations. Optimization prioritized uniform STV prescription coverage while preserving peripheral dose fall‐off; no additional conformity tuning or OAR constraints were introduced because the study focused on intrinsic dose attenuation behavior under standardized stereotactic VMAT conditions.

### 2.4 Dose volume extraction and equivalent radius calculation

For each treatment plan, the volume enclosed by the 15 Gy isodose surface (*V*_15_) was extracted from the three‐dimensional dose distribution. For the 15 Gy × 3 regimens, the 15 Gy isodose corresponded to the prescription surface of the STV and was treated as a geometric reference for modeling purposes. In clinical SCART planning, peripheral dose constraints are commonly considered as safety references; in the present study, the 15 Gy isodose surface was selected as a stable, volume‐preserving geometric surrogate to characterize internal dose attenuation under stereotactic VMAT delivery.

Because the target volumes were constructed as regular cylinders within a homogeneous phantom and were irradiated using coplanar stereotactic VMAT arcs, the resulting dose distributions exhibited approximate axial symmetry around the central axis of the cylinder. Lateral beam modulation predominantly generated approximately symmetric radial dose attenuation around the cylindrical target axis. This geometric symmetry justified the use of a cylindrical equivalent formulation to characterize internal dose fall‐off. Accordingly, the measured 15 Gy isodose volume was converted into an equivalent radial distance using a cylindrical formulation.

The equivalent radius was calculated as:

$$\;r_{15}=\sqrt{\frac{V_{15}}{\pi h}}$$

where *r*_15_ denotes the equivalent radius, *V*_15_ is the volume enclosed by the 15 Gy isodose surface, and *h* is the axial length of the STV. This calculation assumes that the axial extent of the intermediate isodose surface is reasonably comparable to that of the centrally prescribed STV. To assess the validity of this approximation, the axial extent of the measured 15 Gy isodose surface was evaluated in representative geometric configurations and compared with the corresponding STV length. Absolute and relative deviations were subsequently quantified to characterize the magnitude and consistency of the approximation under the investigated stereotactic VMAT conditions. This geometric descriptor provides a consistent framework for relating the centrally prescribed STV to an equivalent outer reference geometry. Here, “equivalent” denotes a volume‐preserving cylindrical approximation used to provide a consistent geometric descriptor rather than an exact physical representation of the isodose surface.

The derived equivalent radius was subsequently used to compare internal dose attenuation across different STV geometries, prescription dose levels, delivery platforms, beam modes, and arc numbers, forming the basis for geometric modeling of the outer reference target–STV scaling relationship.

### 2.5 STV scaling model and inner margin definition

To establish a quantitative relationship between target geometry and internal dose attenuation, the derived *r*_15_ was used as the primary geometric descriptor. For each prescription dose level, linear regression analysis was performed to model the dependence of the predicted STV radius (*d*) on the corresponding target geometry. The corresponding inward scaling distance (inner margin) was subsequently calculated. A linear formulation was selected because exploratory analysis demonstrated an approximately monotonic and near‐linear relationship between predicted STV radius and the investigated geometric descriptors across the evaluated stereotactic configurations.

The predicted STV radius (*d*) was defined as the equivalent radial dimension of the centrally prescribed STV derived from an equivalent outer reference target, characterized by its radius (*c*) and axial length (*b*). In the phantom‐based model, this outer reference target was not explicitly contoured, but its geometry was implicitly inferred from the spatial characteristics of the intermediate isodose surface.

$$d=p_{0}+p_{1}\cdot c+p_{2}\cdot b$$

where *p*_0_, *p*_1_ and *p*_2_ are dose specific fitting coefficients describing the dependence of the predicted STV radius on target geometry. Separate models were generated for each prescription dose to account for differences in dose fall‐off behavior associated with increasing central dose levels. The proposed model was intended as a practical geometric approximation for planning purposes rather than a direct physical dose prediction model.

Based on the fitted STV radius (*d*), the radial contraction required to derive the STV from the GTV was defined as the inner margin.

Innermargin=c−d

This inner margin represents the radial contraction necessary to confine the centrally prescribed high‐dose region within the larger tumor volume while preserving the characteristic internal dose gradient observed under stereotactic VMAT delivery. The resulting scaling approach enables rapid estimation of STV dimensions from GTV geometry and provides a reproducible basis for STV contouring in SCART planning.

### 2.6 Statistical analysis

Geometric and dose‐volume parameters were summarized descriptively. Linear regression models were fitted using the least‐squares method for each prescription dose level. Differences across delivery configurations were evaluated descriptively using absolute differences in *r*_15_. Because simulations were deterministic and did not involve repeated stochastic sampling, formal hypothesis testing was not performed. Data processing and regression analyses were conducted using MATLAB and Microsoft Excel.

### 2.7 Ethical considerations

This study was a phantom‐based dosimetric investigation and did not involve human participants, patient data, or biological specimens. Therefore, institutional review board (IRB) approval and informed consent were not required.

## 3 RESULTS

### 3.1 Geometry‐dependent internal dose fall‐off under stereotactic VMAT delivery

**FIGURE 2:**
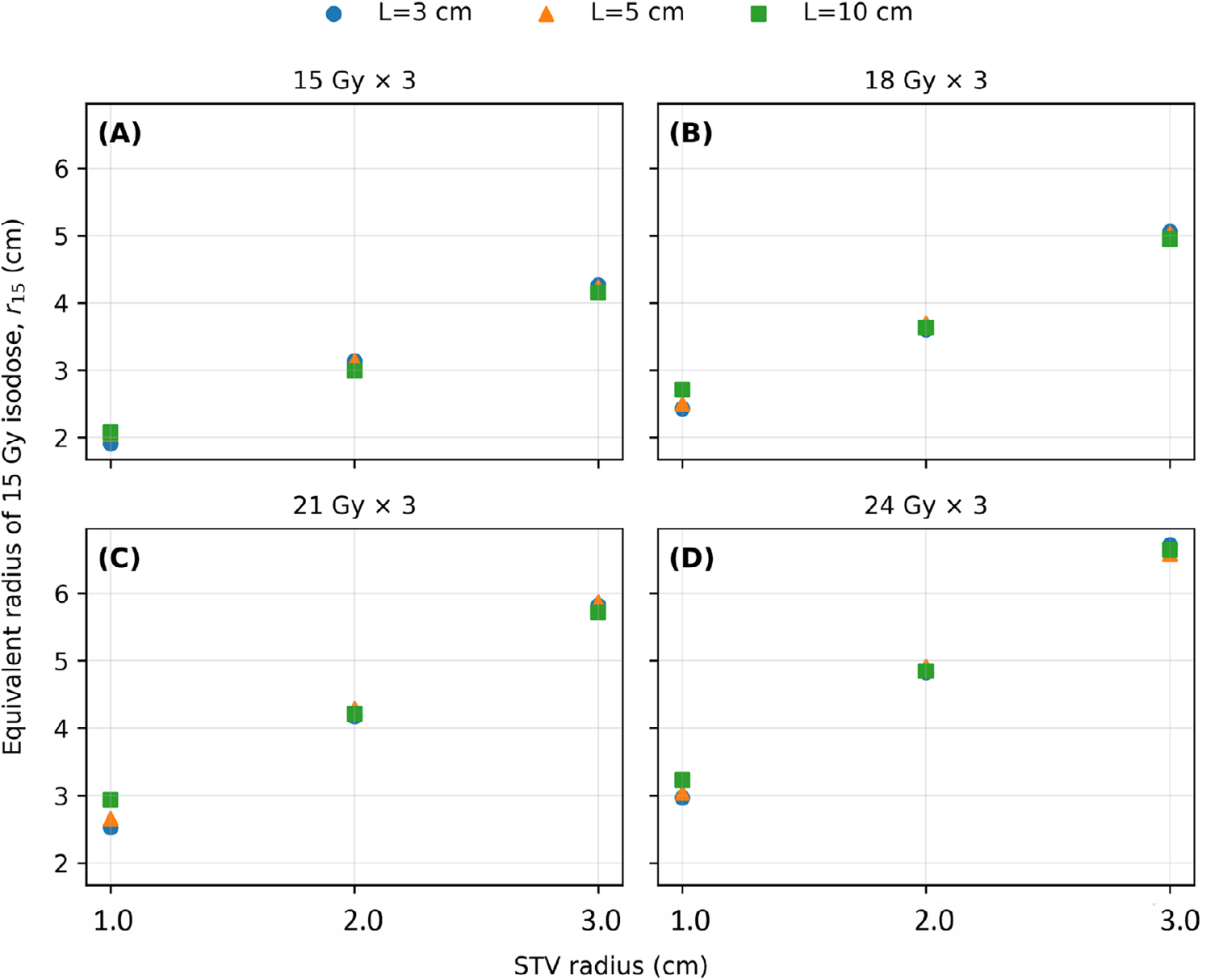
Relationship between STV radius and equivalent radius (*r*_15_) of the 15 Gy isodose surface. Relationship between STV radius and the equivalent radius of the 15 Gy isodose surface (*r*_15_) across different prescription doses. Panels (A)–(D) correspond to prescription regimens of 15 Gy × 3, 18 Gy × 3, 21 Gy × 3, and 24 Gy × 3, respectively. Symbols indicate different STV axial lengths (L = 3, 5, and 10 cm). For each prescription level, *r*_15_ increased monotonically with STV radius and demonstrated limited dependence on axial length.

Representative axial and sagittal dose distributions for cylindrical STVs under stereotactic VMAT delivery are shown in Figure 2. Across all prescription dose levels, the extracted 15 Gy isodose surface remained geometrically consistent across the investigated stereotactic VMAT configurations.

The *r*_15_ increased monotonically with increasing STV radius across all prescription dose levels. For STVs with radii of 1–3 cm, *r*_15_ increased from approximately 2.0–2.1 cm to 4.1–4.3 cm at 15 Gy × 3, and from approximately 3.0–3.3 cm to 6.3–6.6 cm at 24 Gy × 3. Increasing prescription dose produced systematic outward displacement of the intermediate isodose surface while preserving the overall geometric relationship between *r*_15_ and STV size. Accordingly, the relatively larger *r*_15_ values observed in the 18 Gy × 3 group compared with the 15 Gy × 3 reference condition are expected because the 15 Gy isodose represents a lower intermediate dose level for higher prescription regimens.

At fixed STV radius, the influence of axial length was comparatively small. Across STV lengths of 3–10 cm, variation in *r*_15_ generally remained within approximately 0.2–0.4 cm for a given prescription level, indicating that radial geometry was the dominant determinant of internal dose attenuation behavior (Supplementary Figure S1). Representative comparisons demonstrated systematic axial extension of the 15 Gy isodose surface beyond the nominal STV length, with relative deviations ranging from approximately 14% to 50% depending on target size and prescription level (Supplementary Table S1).

For the 15 Gy × 3 regimens, the 15 Gy isodose coincided with the prescription surface and was therefore treated as a geometric reference condition. Although minor axial extension of the isodose surface was observed, the equivalent‐radius formulation remained applicable across the investigated geometries and prescription levels.

### 3.2 Influence of arc number on internal dose attenuation

**FIGURE 3:**
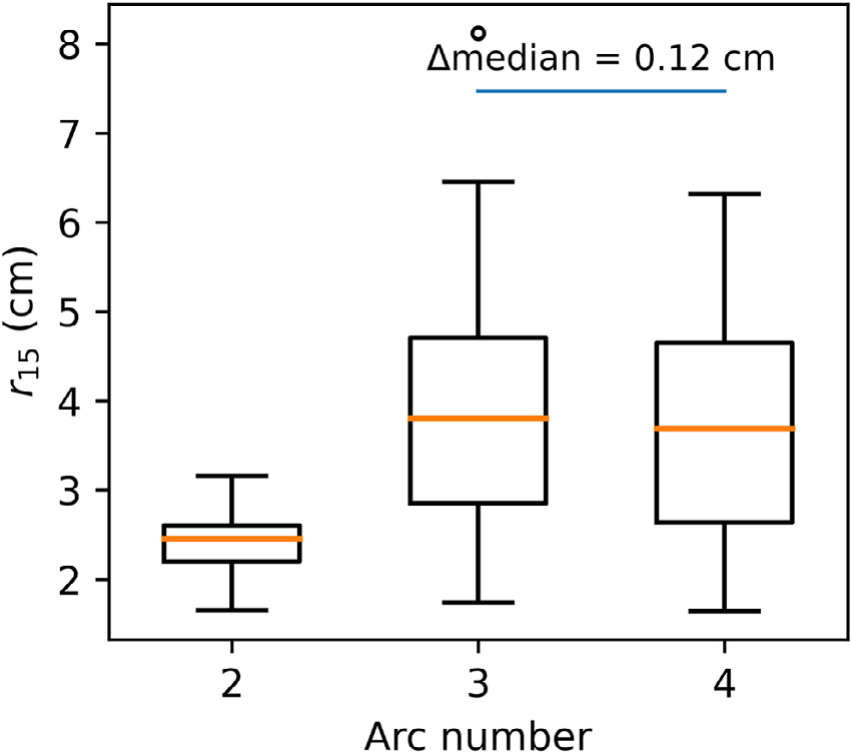
Distribution of equivalent radius (*r*_15_) according to VMAT arc number. Boxplots summarize the distribution of *r*_15_ values across representative stereotactic VMAT configurations using 2‐, 3‐, and 4‐arc delivery techniques. The central line represents the median; box boundaries indicate the interquartile range (IQR); whiskers indicate the full observed data range excluding outliers. Median *r*_15_ values remained comparable across delivery techniques, with a median inter‐configuration difference of approximately 0.12 cm.

Increasing arc number produced slightly steeper internal dose gradients, reflected by modest reductions in the radial extent of the intermediate isodose surface. Median differences in *r*_15_ between arc configurations remained approximately 0.12 cm, indicating that target geometry remained the predominant determinant of internal dose fall‐off. Although 2‐arc plans generally exhibited slightly larger *r*_15_ values, substantial overlap in *r*_15_ distributions was observed across delivery configurations (Figure 3).

### 3.3 Geometry‐based STV scaling model

The fitted parameters of the geometry‐based STV scaling model for each prescription dose are summarized in Table 1. Across all evaluated prescription levels, internal dose fall‐off behavior could be consistently described using a three‐parameter linear formulation relating predicted STV radius (*d*) to the outer reference geometry parameters (*c*) and (*b*).

**TABLE 1:** STV scaling model parameters (*p_0_*, *p_1_*, *p_2_*) derived for each prescription dose.

Prescription dose (Gy)	*p*_0_	*p*_1_	*p*_2_
15	−0.3579	0.4410	0.0110
18	0.1784	0.0042	0.1345
21	0.1754	0.0041	0.1344
24	0.1841	0.0015	0.1345

The predicted STV radius (d) was modeled as: $d=p_{0}+p_{1}\cdot c+p_{2}\cdot b$.

The inward scaling distance was subsequently calculated as (*c* − *d*). For the 15 Gy prescription, the 15 Gy isodose coincides with the prescription surface and serves as a geometric reference. Model parameters were fitted separately for each prescription dose. All distances are reported in centimeters.

The fitted regression surfaces demonstrated near‐linear relationships between predicted STV radius and geometric descriptors across all prescription levels (Supplementary Figure S2). Regression analysis demonstrated excellent agreement between the fitted geometric models and the phantom‐derived datasets, with R^2^ values ranging from 0.991 to 0.999 across prescription levels. To further characterize model accuracy, RMSE and MAE were calculated for each prescription‐specific model (Supplementary Table S2). RMSE values ranged from 0.014 to 0.038 cm and MAE values ranged from 0.011 to 0.031 cm, indicating low absolute prediction errors across the investigated geometric configurations. While the intercept term varied according to prescription dose, the geometric coefficients remained relatively stable for prescription levels of 18 Gy and above, indicating preservation of the overall scaling relationship despite systematic shifts in dose attenuation behavior. For the 15 Gy × 3 regimens, the fitted coefficients represent a geometric reference scenario in which the 15 Gy isodose coincides with the prescription surface, resulting in stronger apparent dependence on the radial geometry parameter. In contrast, for higher prescription doses, the 15 Gy isodose represented an intermediate internal dose level within the target volume, leading to more consistent geometry‐dependent attenuation behavior across configurations.

### 3.4 Representative clinical implementation of the STV scaling framework

**FIGURE 4:**
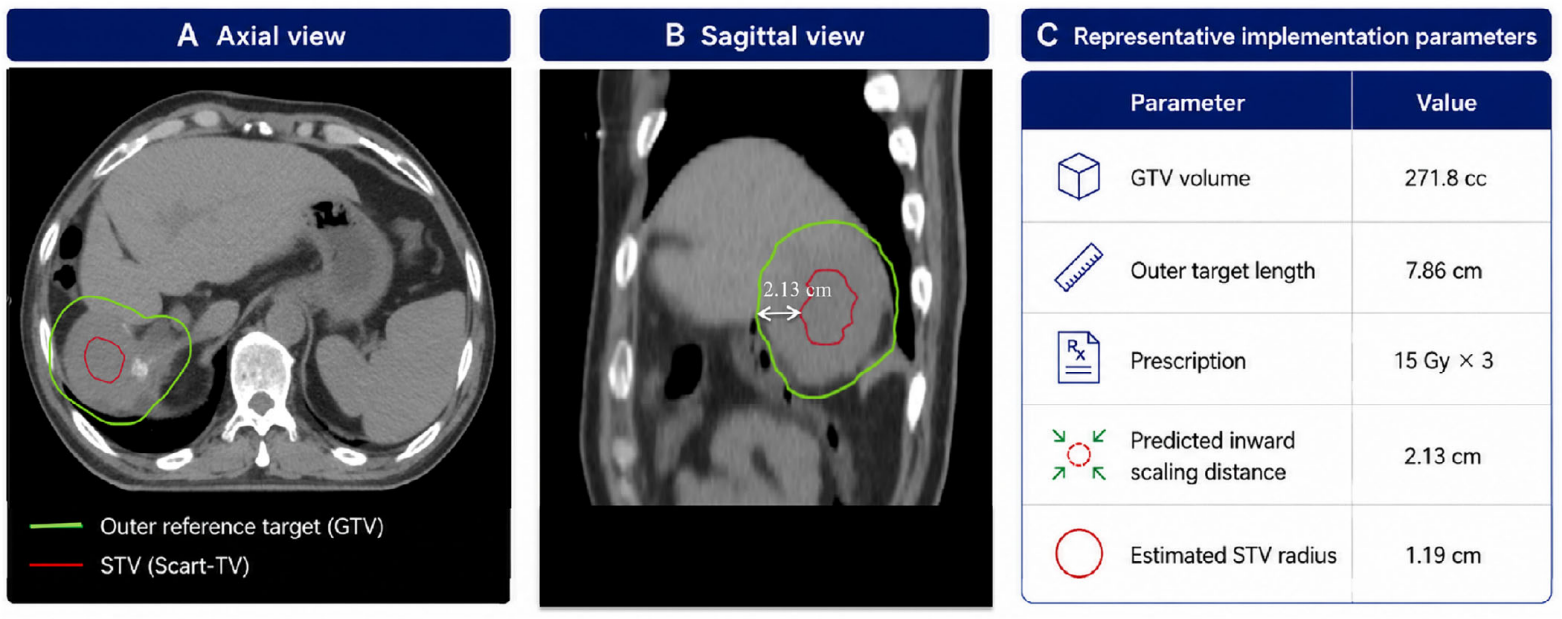
Representative clinical implementation of the geometry‐based STV scaling framework. Axial (A) and sagittal (B) planning CT views illustrating inward scaling from an outer reference target (green) to the derived STV (red) in a representative liver lesion. The corresponding geometric parameters, model‐predicted STV radius, and derived inward scaling distance are summarized in Panel C.

To illustrate potential clinical implementation, a representative liver SCART planning case was retrospectively evaluated using the proposed geometry‐based scaling model (Figure 4). The GTV volume was 271.8 cc with an axial length of 7.86 cm. Using the 15 Gy × 3 scaling model, the predicted STV radius was 1.19 cm, corresponding to an inward contraction distance of 2.13 cm from the outer reference target. To further evaluate the applicability of the proposed framework across a broader range of clinical scenarios, four additional validation cases involving different disease sites and target geometries were analyzed. The corresponding geometric parameters and model‐derived scaling results are summarized in Supplementary Table S3.

The resulting STV remained concentrically confined within the outer reference target and was geometrically consistent with the centrally prescribed high‐dose region. Axial and sagittal views showed the expected internal dose attenuation pattern, with smooth dose fall‐off extending toward the peripheral tumor region. The representative implementation example demonstrated that the proposed scaling framework may be incorporated into a clinical stereotactic planning workflow using measurable geometric parameters derived from routine target delineation.

## 4 DISCUSSION

In this study, we developed a geometry‐based STV scaling framework for SCART using controlled phantom‐based stereotactic VMAT simulations. The observed reproducibility of internal dose fall‐off across target geometries supports the feasibility of inward STV scaling under controlled stereotactic VMAT conditions.

A principal finding of this work is that internal dose fall‐off under stereotactic VMAT delivery was predominantly associated with target geometry rather than delivery configuration. Across all investigated prescription levels, *r*_15_ increased monotonically with target size while remaining comparatively stable across beam modes and arc arrangements. Variation associated with axial target length generally remained within approximately 0.2–0.4 cm, whereas median inter‐configuration differences across arc numbers remained approximately 0.12 cm. Similar target‐size‐dependent dose fall‐off behavior has been reported in previous stereotactic planning studies.^17^, ^18^ These observations support the use of reproducible geometric descriptors for inward STV scaling without repeated plan optimization or subjective visual adjustment.

The optimal prescription and fractionation strategy for SCART remains uncertain.^19^ In the present study, fitted scaling coefficients remained relatively consistent for prescription levels of 18 Gy and above, suggesting reduced sensitivity of the attenuation pattern to further prescription dose escalation. Regression models demonstrated excellent agreement with phantom‐derived measurements (R^2^ = 0.991–0.999), supporting the reproducibility of the proposed scaling relationship under the investigated stereotactic VMAT conditions. The observed axial deviations (approximately 14–50%) reflect an inherent limitation of the equivalent cylindrical approximation used to characterize the 15 Gy isodose surface. Although the equivalent‐radius formulation preserves the overall enclosed volume, it does not explicitly reproduce longitudinal variations of the actual three‐dimensional isodose distribution. Consequently, differences in axial extent are expected when a complex stereotactic dose distribution is represented using a simplified cylindrical geometry. Importantly, the proposed framework was developed to estimate the STV radius and corresponding inward scaling distance required for STV generation. Therefore, although axial deviations were observed, they are unlikely to substantially affect the intended geometric scaling application of the model. Nevertheless, prospective clinical validation remains necessary.

From a clinical implementation perspective, the principal contribution of this study lies in providing a rapid and reproducible approach for STV generation based on measurable GTV geometry. By translating internal dose attenuation characteristics into a regression‐based geometric scaling relationship, the proposed model enables estimation of STV radius without extensive trial‐and‐error optimization. Importantly, the model‐derived STV should be regarded as a planning reference rather than a definitive contour.^20^, ^21^ Physician review and manual modification remain necessary, particularly for tumors with irregular morphology, marked concavities, or close proximity to critical organs, where purely geometry‐based inward contraction may not adequately reflect clinically relevant dose–volume trade‐offs.

The proposed framework differs from existing lattice or spatially fractionated radiotherapy approaches, in which high‐dose subvolumes are typically generated using predefined spatial patterns or planner‐dependent optimization strategies.^22^ In contrast, the present framework derives inward STV scaling directly from reproducible VMAT‐derived internal dose attenuation behavior as a function of target geometry. Previous geometry‐ or dose‐volume‐based modeling studies have primarily focused on dose prediction or treatment evaluation within established radiotherapy paradigms, including partial stereotactic ablative boost radiotherapy (P‐SABR) approaches for bulky tumors^23^ and geometry‐associated dose prediction models for bulky tumor radiotherapy.^24^ The principal contribution of the present work therefore lies not in geometry‐based modeling itself, but in translating reproducible VMAT‐derived dose attenuation characteristics into a practical framework for prospective STV definition in SCART planning. Unlike previous geometry‐based prediction models that primarily estimated dosimetric outcomes after treatment planning, the present framework was specifically developed to support prospective geometric STV construction before optimization during SCART planning.

A representative liver SCART planning case was retrospectively evaluated to illustrate potential clinical implementation. The model‐derived STV remained concentrically located within the outer reference target and preserved the characteristic internal dose attenuation pattern observed in the phantom analysis. This representative implementation should therefore be interpreted as a preliminary geometry‐guided planning demonstration rather than a clinically validated STV definition strategy.

Several limitations should be acknowledged. First, the proposed framework was derived using homogeneous phantom geometries under controlled coplanar VMAT conditions and therefore does not account for tissue heterogeneity, respiratory motion, or non‐coplanar delivery techniques encountered clinically. Second, idealized cylindrical targets facilitated controlled geometric analysis but do not fully represent the morphological diversity of clinical tumors. Third, only the 15 Gy intermediate isodose surface was evaluated as the reference condition, and alternative intermediate dose levels may exhibit different attenuation characteristics. Finally, clinical implementation was demonstrated using only a single retrospective illustrative case. Therefore, the proposed model should currently be regarded as a proof‐of‐concept geometry‐based planning approximation rather than a clinically validated STV definition strategy.

## 5 CONCLUSION

This study presents a reproducible framework for STV definition in SCART based on controlled phantom‐derived stereotactic VMAT dose distributions. Internal dose fall‐off demonstrated a stable target‐size‐associated pattern that could be quantitatively characterized using an equivalent‐radius formulation.

Based on these observations, a dose‐specific geometric scaling model was developed to relate outer target dimensions to the predicted STV radius and corresponding contraction distance. The resulting regression‐based framework provides a practical reference for inward STV scaling and may support more standardized SCART planning. Although derived under simplified phantom conditions, the proposed approach establishes a physically interpretable basis for STV definition. Further validation in heterogeneous anatomical environments and larger clinical cohorts will be required before broader clinical implementation.

## AUTHOR CONTRIBUTIONS

**Ningjing Siah**: Visualization; writing—original draft; writing—review & editing. **Pengyuan Qi**: Formal analysis; writing—original draft. **Bin Hu**: Data curation; writing—review & editing. **Mi Chen**: Methodology; investigation. **Ting Cao**: Methodology; data curation. **Xin Li**: Conceptualization; supervision. **Jun Han**: Conceptualization; supervision.

## CONFLICT OF INTEREST STATEMENT

The authors declare that they have no conflicts of interest.

## Supporting information



Supporting Information

## Data Availability

The data that support the findings of this study are available from the corresponding author upon reasonable request.
