# Supplementary material for: A geometry‐based scaling model for stereotactic treatment volume definition in stereotactic centralized ablative radiation therapy
Source: J Appl Clin Med Phys. 2026 Jul 26;27(8):e70713. doi: 10.1002/acm2.70713 (PMC13402241; doi:10.1002/acm2.70713)
Supplement: Supplementary file 1 — Supporting Information [file ACM2-27-e70713-s001.docx]

**
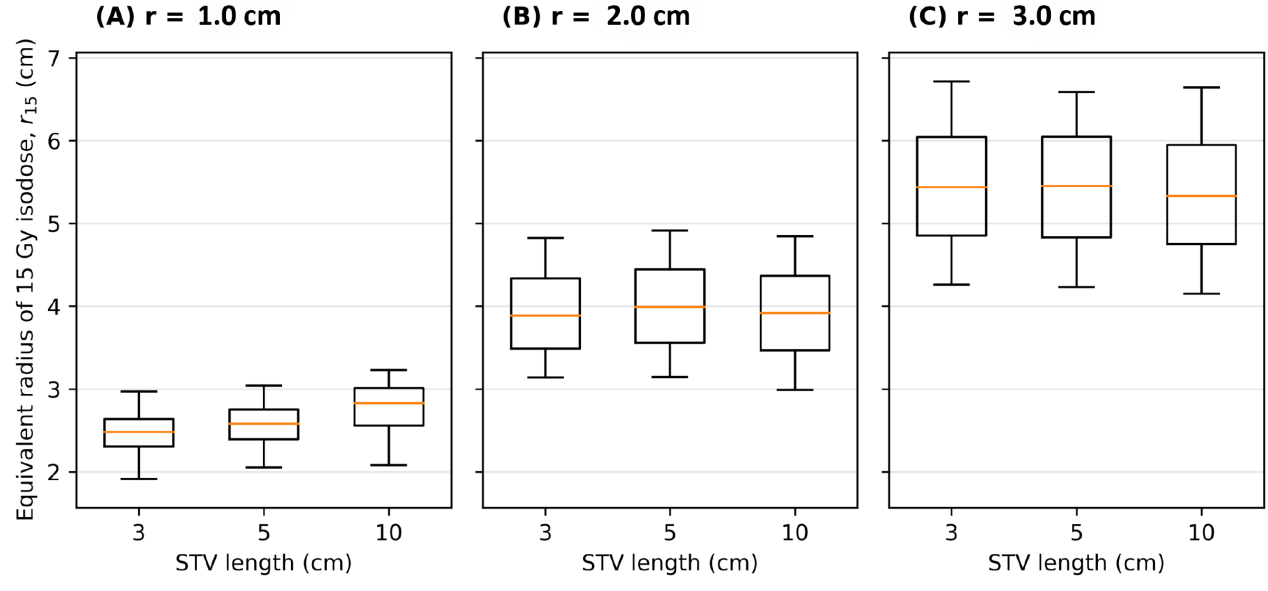
**

**Figure S1. Distribution of equivalent radius (*r*_15_) across different STV lengths for representative target radii.**

Distribution of the equivalent radius of the 15 Gy isodose surface (*r*_15_) as a function of STV axial length for three STV radii: (A) r = 1.0 cm, (B) r = 2.0 cm, and (C) r = 3.0 cm. Boxplots summarize the variation of *r*_15_ across axial lengths of 3, 5, and 10 cm, pooled over prescription doses and arc configurations. Variation in *r*_15_ associated with axial length remained comparatively limited across the investigated configurations. In each boxplot, the central line indicates the median, box boundaries represent the interquartile range (IQR), and whiskers indicate the full observed data range excluding outliers.

**Table S1. Comparison between nominal STV axial length and the measured axial extent of the 15 Gy isodose surface under representative stereotactic VMAT configurations.**

| Nominal STV axial length (cm) | Measured axial extent of 15 Gy isodose surface (cm) | Absolute deviation (cm) | Relative deviation (%) |
| --- | --- | --- | --- |
| 3 | 4.05–4.50 | 1.05–1.50 | 35.0–50.0 |
| 5 | 6.20–6.50 | 1.20–1.50 | 24.0–30.0 |
| 10 | 11.40–12.00 | 1.40–2.00 | 14.0–20.0 |
| Measured axial extent refers to the superior–inferior length of the extracted 15 Gy isodose surface. Absolute deviation was calculated as the difference between the measured axial extent of the 15 Gy isodose surface and the nominal STV axial length. Relative deviation was calculated as the percentage difference relative to the nominal STV axial length. Representative geometric configurations were selected to illustrate the magnitude of axial extension under controlled stereotactic VMAT conditions. | | | |


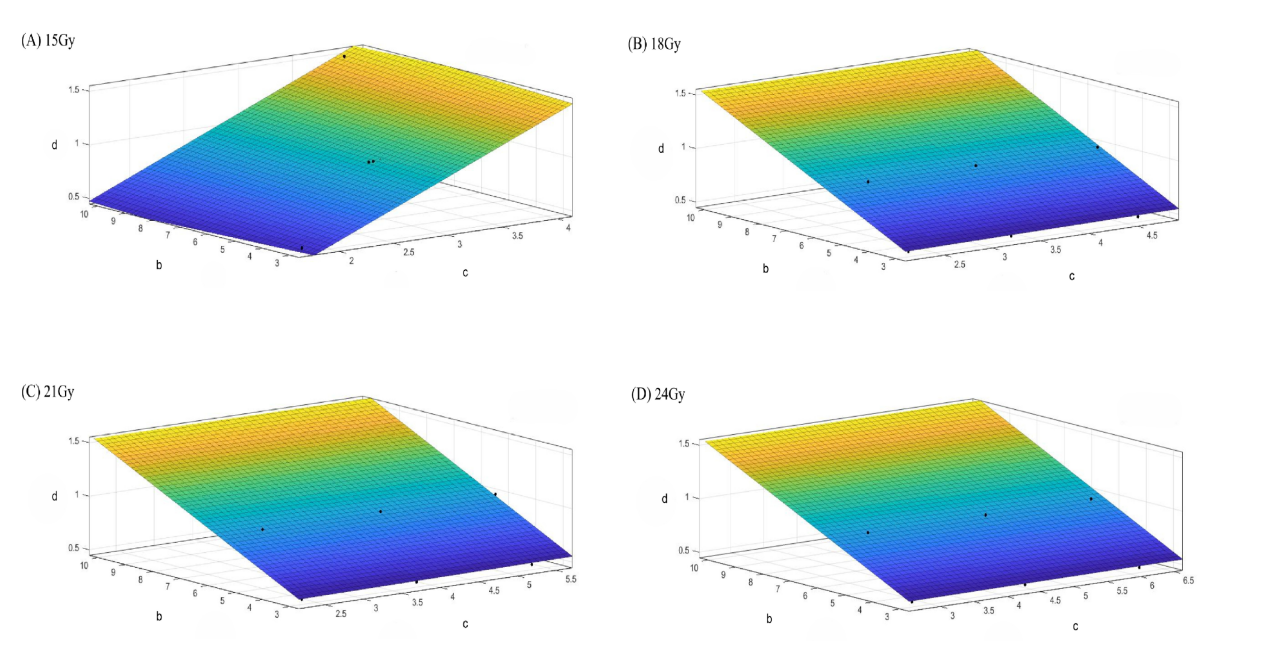


**Figure S2.**

Three-dimensional fitted surfaces illustrating the relationship between the predicted STV radius (*d*) and the geometric parameters *c* (outer reference radius) and *b* (axial length) for each prescription dose level: (A) 15 Gy× 3, (B) 18 Gy× 3, (C) 21 Gy× 3, and (D) 24 Gy× 3. Black dots represent measured data points derived from phantom-based stereotactic VMAT simulations, and the surfaces represent the corresponding dose-specific linear regression models.

**Table S2. Performance metrics of the dose-specific geometry-based STV scaling models.**

| Prescription dose | R² | RMSE (cm) | MAE (cm) | Max absolute error (cm) |
| --- | --- | --- | --- | --- |
| 15 Gy × 3 | 0.995 | 0.03 | 0.026 | 0.049 |
| 18 Gy × 3 | 0.991 | 0.038 | 0.031 | 0.065 |
| 21 Gy × 3 | 0.994 | 0.03 | 0.027 | 0.044 |
| 24 Gy × 3 | 0.999 | 0.014 | 0.011 | 0.03 |
| R² represents the coefficient of determination. RMSE and MAE were calculated from the differences between observed and model-predicted STV radii (*d*). All error metrics are reported in centimeters. Lower RMSE and MAE values indicate improved predictive accuracy of the scaling model. | | | | |

**Table S3. Additional validation cases**

| Case | Site | GTV Volume (cc) | Length (cm) | Prescription | Predicted STV radius (cm) | Inward scaling distance (cm) |
| --- | --- | --- | --- | --- | --- | --- |
| 1 | Liver | 271.8 | 7.86 | 15 Gy × 3 | 1.19 | 2.13 |
| 2 | Liver | 1015.7 | 11.25 | 15 Gy × 3 | 2.13 | 3.23 |
| 3 | Lung | 108.7 | 5.2 | 15 Gy × 3 | 0.84 | 1.74 |
| 4 | Pelvic | 467.3 | 10.43 | 15 Gy × 3 | 1.42 | 2.35 |
| *Abbreviations*: GTV, gross tumor volume; STV, stereotactic treatment volume.  Length represents the measured long-axis dimension of the outer reference target. Predicted STV radius was calculated using the dose-specific scaling model. The corresponding inward scaling distance was subsequently calculated as (*c* − *d*), where *c* is the equivalent radius of the outer reference target and *d* is the model-predicted STV radius. | | | | | | |
